# Polymorphous low-grade adenocarcinoma: an analysis of epidemiological studies and hints for pathologists

**DOI:** 10.1186/1746-1596-8-6

**Published:** 2013-01-15

**Authors:** Vera Cavalcanti de Araujo, Fabricio Passador-Santos, Cecilia Turssi, Andresa Borges Soares, Ney Soares de Araujo

**Affiliations:** 1 Department of Oral Pathology, Sao Leopoldo Mandic Institute and Research Center, Rua Jose Rocha Junqueira13 Ponte Preta, Campinas, SP, 13045-755, Brazil

**Keywords:** Polymorphous low-grade adenocarcinoma, Epidemiological review, Diagnosis hints

## Abstract

**Background:**

This study is an analysis of the prevalence of polymorphous low grade adenocarcinoma (PLGA) in epidemiological surveys of salivary tumors published in the English language from 1992 to 2012.

**Methods:**

These surveys included studies from different researchers, countries and continents. The 57 surveys for which it was possible to calculate the percentage of PLGAs among all malignant minor salivary gland tumors (MMSGT) were included in this review.

**Results:**

The statistical analyses show significant differences in the PLGA percentage by time period, country and continent in the studies included in this review. The percentage of PLGAs among MMSGTs varied among the studies, ranging from 0.0% to 46.8%. PLGA rates have varied over the period studied and have most recently increased. The frequency of reported PLGA cases also varied from 0.0% to 24.8% by the country in which the MMSGT studies were performed. The PLGA percentages also varied significantly by continent, with frequencies ranging from 3.9% in Asia to 20.0% in Oceania

**Conclusion:**

Based on these results, we concluded that although the accuracy of PLGA diagnoses has improved, they remain a challenge for pathologists. To facilitate PLGA diagnoses, we have therefore made some suggestions for pathologists regarding tumors composed of single-layer strands of cells that form all of the histological patterns present in the tumor, consistency of the cytological appearance and uniformly positive CK7, vimentin and S100 immunohistochemistry, which indicate a single PLGA phenotype.

**Virtual slide:**

The virtual slide(s) for this article can be found here: http://www.diagnosticpathology.diagnomx.eu/vs/1059098656858324

## Introduction

Polymorphous low-grade adenocarcinoma (PLGA) is a malignant epithelial tumor characterized by cytological uniformity, morphological diversity, an infiltrative growth pattern and low metastatic potential [1]. This tumor was recognized as a distinct entity in 1983 by Freedman and Lumerman and Batsakis et al., and it was named polymorphous low-grade adenocarcinoma by Evans and Batsakis in 1984 [2-4].

Clinically PLGA presents as an indolent asymptomatic swelling but occasionally can be painful and even ulcerate. The most common location of PLGA is the palate, although other locations have been described. It occurs more frequently in women affecting mainly the sixth and seventh decade of life. For more details on clinical presentation, prognosis and treatment, we recommend the reviews by Pogodzinski et al., and Paleri, Robinson and Bradley [5,6]. In general these authors indicate a low grade malignancy and good prognosis of this tumor They also recommend a very careful and systematic follow- up,since recurrences and rare metastases can occur many years after the surgery.

The tumor is characterized by single-layer strands of cells that can form lobular, tubular, cribriform, trabecular, papillary-cystic and cystic histological patterns, which can be illustrated by the presence of extracellular matrix between the strands of cells identified in lobular or solid patterns [7].

Most PLGA cells are cytologically uniform and range from small to medium in size, with vesicular oval nuclei and inconspicuous nucleoli. Their cytoplasm is ample and exhibits a variable appearance, including eosinophilic, basophilic and clear aspects. The cells have indistinct outlines that lend a syncytial pattern to the active cellular mass. Groups of cells with a coarsely eosinophilic granular cytoplasm, mimicking oncocytes, are occasionally observed, as are mucous cells [1,8].

The cells show a unique electron microscopy and immunohistochemical phenotype. All cells have microvilli apically and are attached to the basal lamina. The cells are positive for vimentin, CK 7 and S100, a pattern only shared by the mammary analogue secretory carcinoma, as recently described by Skalova et al. and rarely by focal plasmacytoid cells in pleomorphic adenoma [8-10]. A regular distribution of positive staining for β1, β2 and β3 integrins and striking bipolar staining in all of the neoplastic cells reinforces this unique phenotype [11].

Single cells, usually infiltrating surrounding structures, and clear cells in nests are also observed in the lobular PLGA subtype. The stroma appears either strongly eosinophilic and hyalinized, or muco-hyalinized with a bluish tint. Foci of residual salivary gland acini surrounded by neoplastic cells are occasionally found. Peri-neural invasion by groups of tumor cells is a frequent finding, and psammoma-like structures are occasionally observed. This tumor frequently presents with prominent vascularity [8].

Despite greater understanding of this tumor, PLGA remains a diagnostic challenge for pathologists. This conclusion is based on the variability of the epidemiological results obtained by several groups who have studied this tumor.

We have reviewed the epidemiological studies in an attempt to analyse the proportion of PLGAs in salivary gland tumors.

## Materials and methods

This analysis included 57 epidemiological studies of salivary gland tumors published in the English language from 1992 to 2012. The year 1992 marked the inclusion of PLGA in the World Health Organization (WHO) classification of salivary gland tumors [12]. Studies were included in the analysis if they contained the data needed to calculate the fraction of PLGAs in the malignant minor salivary gland tumor (MMSGT) total.

The studies addressing only major salivary gland tumors were excluded because the few cases published on that topic do not significantly contribute to the understanding of PLGA; similarly, studies that included only children and adolescents were excluded from this analysis.

When data were available, we extracted the following information: total number of salivary gland tumors; number of minor salivary gland tumors and their fraction of the total number of tumors; number of MMSGTs and their fraction of the total number of minor salivary gland tumors; number of PLGAs and their fraction of the total number of MMSGTs; and the total number of minor salivary gland tumors.

In this study, we analyzed the fraction of PLGAs in the total number of MMSGTs. It was not possible to obtain the absolute or relative frequencies of malignant salivary tumors, either among minor salivary tumors alone or among major and minor tumors, from studies that reported only MMSGTs.

### Statistical analysis

Data were tabulated and descriptive statistics were calculated using frequency tables. G tests were used to ascertain whether the PLGA fraction of all MMSGTs varied by the year, country and continent in which the studies were performed. We would like to emphasize that at no point was it presumed that these studies reflect the prevalence of this tumor with respect to the aforementioned variables (year, country and continent). The significance level was set at 5%. The statistical calculations were performed using the SPSS 20 software package (IBM corporation, Armonk, NY, USA).

## Results

Fifty-seven surveys of salivary gland tumors were included in this review (Table 1) [13-68]. From 26,960 cases of salivary gland tumors, 431 (1,6%) were accepted by the authors as been PLGAs.

**Table 1:** The distribution of salivary gland tumors, minor salivary gland tumors and polymorphous low-grade adenocarcinoma in the studies included in this review

**Author**	**Year**	**Country**	**SGTs**	**MSGTs**		**MMSGTs**		**PLGAs**	
			**n**	**n**	**% in relation to SGTs**	**n**	**% in relation to Minor SGTs**	**n**	**% in relation to MMSGTs**
Onyango et al.	1992	Kenya	417	189	45.3	58	30.7	0	—
Rippin e Potts	1992	England	194	194	—	88	45.4	0	—
Loyola et al.	1995	Brazil	164	164	—	65	39.6	4	6.2
Neely et al.	1996	USA	106	106	—	47	44.3	22	46.8
Rivera-Bastidas et al.	1996	Venezuela	62	62	—	28	45.2	0	—
Rushing et al.	1996	USA	277	27	9.7	16	59.3	0	—
Kusama et al.	1997	Japan	129	129	—	49	38.0		—
Nagler et al.	1997	Israel	245	67	27.3	33	49.3	3	9.1
Jones et al.	1998	England	145	145	—	103	71.0		—
Lopes et al.	1999	Brazil	196	196	—	129	65.8	3	2.3
Maaita et al.	1999	Jordan	221	42	19.0	20	47.6	0	—
Pacheco-Ojeda et al.	2000	Ecuador	308	28	9.1	14	50.0	0	—
Koivunen et al.	2002	Finland	40	4	10.0	4	—	0	—
Vargas et al.	2002	Brazil	124	6	4.8	4	66.7	0	—
Masanja et al.	2003	Tanzania	153	66	43.1	37	56.1	0	—
Hyan et al.	2004	Australia	30	30	—	30	—	6	20.0%
Kokemueller et al.	2004	German	155	90	58.1	90	—	7	7.8
Poomsawat et al.	2004	Thailand	60	54	90.0	37	68.5	1	2.7
Strick	2004	England	21	21	—	21	—	5	23.8
Toida et al.	2004	Japan	82	82	—	27	32.9	0	—
Vuhahula	2004	Uganda	268	88	32.8	47	53.4	7	14.9
Lima et al.	2005	Brazil	245	46	18.8	22	47.8	0	—
Ito et al.	2005	Brazil	496	113	22.8	53	46.9	9	17.0
Luukkaa et al.	2005	Finland	46	46	—	46	—	8	17.4
Otho et al.	2005	Niger	79	33	41.8	14	42.4	0	—
Yih et al.	2005	USA	213	213	—	94	44.1	18	19.1
Ascani et al.	2006	Italy	454	30	6.6	7	23.3	0	—
Ansari et al.	2007	Iran	130	18	13.8	16	88.9	0	—
Buchner et al.	2007	USA	380	380	—	156	41.1	27	17.3
Jones et al.	2007	England	741	455	61.4	172	37.8	28	16.3
Ladeinde et al.	2007	Niger	120	76	63.3	52	68.4	5	9.6
Pires et al.	2007	USA	546	546	—	241	44.1	28	11.6
Wang et al.	2007	China	737	737	—	397	—	34	8.6
Copeli et al.	2008	Italy	43	43	—	43	—	1	2.3
Li et al.	2008	China	3,461	914	26.4	539	59.0	1	0.2
Rahman et al.	2008	Paquistan	70	70	—	70	—	2	2.9
Subhashraj et al.	2008	India	684	150	21.9	59	39.3	0	—
Chijiwa et al.	2009	Japan	22	22	—	22	—	0	—
Dhanuthai	2009	Thailand	311	311	—	164	52.7	2	1.2
Gao et al.	2009	China	1,062	519	48.9	519	—	19	3.7
Mucke et al.	2009	German	95	95	—	95	—	14	14.7
Ochicha et al.	2009	Niger	78	19	24.4	7	36.8	2	28.6
Oliveira et al.	2009	Brazil	599	87	14.5	50	57.5	0	—
Targa-Stramandinoli et al.	2009	Brazil	14	14	—	7	50.0	1	14.3
Tilakaratne et al.	2009	Sri Lanka	713	486	68.2	276	56.8	27	9.8
Carrillo et al.	2010	Mexico	77	77	—	77	—	0	—
Erovic et al.	2010	Austria	32	32	—	32	—	0	—
Kakarala & Bhattacharyya	2010	USA	639	639	—	639	—	0	—
Kruse et al.	2010	Switzerland	27	27	—	27	—	0	—
Tian et al.	2010	China	6,982	1,977	28.3	1228	62.1	29	2.4
Bjorndal et al.	2011	Denmark	952	266	27.9	266	—	66	24.8
Morais et al.	2011	Brazil	303	37	12.2	26	70.3	3	11.5
Schwarz et al.	2011	German	41	41	—	41	—	8	19.5
Venkata et al.	2011	India	185	185	—	138	74.6	18	13.0
Bello et al.	2012	Finland	1,888	177	9.4	68	38.4	11	16.2
Bello et al.	2012	Israel	330	111	33.6	71	64.0	8	11.3
Luksic et al.	2012	Croatia	768	297	38.7	210	70.7	4	1.9
Total	—	—	26,960	11,079	41.1	6,891	62.2	431	6.3

SGTs: salivary gland tumors; MSGTs minor salivary gland tumors; MMSGTs: malignant minor salivary gland tumors; PLGA: polymorphous low-grade adenocarcinoma.

There has been a significant increase (p < 0.0001 for the G test) in the fraction of PLGA cases reported in the literature since 2007, as shown in Table 2. Epidemiological studies from 1992 to 1994 and 2001 to 2003 included no reports of PLGAs, whilst 1.8% of the MMSGTs reported from 1998 to 2000 were PLGAs. Higher percentages were noted from 1995 to 1997 and 2007 to 2012. The highest PLGA percentages were reported in the studies published from 2004 to 2006 (Table 2).

**Table 2:** The numbers and percentages of polymorphous low-grade adenocarcinomas in malignant minor salivary gland tumors by publication year as described in the studies included in this review

** Year**	**MMSGTs**		**PLGA**		**PLGA/MMSGTs**
	**n**	**%**	**n**	**%**	**%**
1992-1994	146	2.1	0	0.0	0.0
1995-1997	341	4.9	29	6.7	8.5
1998-2000	163	2.4	3	0.7	1.8
2001-2003	45	0.7	0	0.0	0.0
2004-2006	488	7.1	61	14.2	12.5
2007-2009	2,885	41.9	191	44.3	6.6
2010-2012	2,823	41.0	147	34.1	5.2
** Total**	**6,891**	**100.0**	**431**	**100.0**	**6.3**

MMSGTs: malignant minor salivary gland tumors; PLGA: polymorphous low-grade adenocarcinoma.

The frequency of PLGA also varied significantly (p < 0.0001 for the G test) by country, as shown in Table 3. Of the 431 PLGA cases included in this review (Table 1), 95 (22.0%) were from studies performed in the USA, 83 (19.3%) were from Chinese studies and 66 (15.3%) were from Danish studies. The percentage of PLGAs among MMSGTs varied among the studies, ranging from 0.0% to 24.8% (Table 3).

**Table 3:** The numbers and percentages of polymorphous low-grade adenocarcinomas in malignant minor salivary gland tumors by country as described in the studies included in this review

**Country**	**MMSGTs**		**PLGA**		**PLGA/MMSGTs**
	**n**	**%**	**n**	**%**	**%**
Australia	30	0.4	6	1.4	20.0
Austria	32	0.5	0	0.0	0.0
Brazil	356	5.2	20	4.6	5.6
China	2,683	38.9	83	19.3	3.1
Croatia	210	3.0	4	0.9	1.9
Denmark	266	3.9	66	15.3	24.8
Ecuador	14	0.2	0	0.0	0.0
England	384	5.6	33	7.7	8.6
Finland	118	1.7	19	4.4	16.1
German	226	3.3	29	6.7	12.8
India	197	2.9	18	4.2	9.1
Iran	16	0.2	0	0.0	0.0
Israel	104	1.5	11	2.6	10.6
Italy	50	0.7	1	0.2	2.0
Japan	98	1.4	0	0.0	0.0
Jordan	20	0.3	0	0.0	0.0
Kenya	58	0.8	0	0.0	0.0
Mexico	77	1.1	0	0.0	0.0
Niger	73	1.1	7	1.6	9.6
Paquistan	70	1.0	2	0.5	2.9
Sri Lanka	276	4.0	27	6.3	9.8
Switzerland	27	0.4	0	0.0	0.0
Tanzania	37	0.5	0	0.0	0.0
Thailand	201	2.9	3	0.7	1.5
Uganda	47	0.7	7	1.6	14.9
USA	1,193	17.3	95	22.0	8.0
Venezuela	28	0.4	0	0.0	0.0
**Total**	**6,891**	**100.0**	**431**	**100.0**	**6.3**

MMSGTs: malignant minor salivary gland tumors; PLGA: polymorphous low-grade adenocarcinoma.

The frequency of reported PLGA cases also varied significantly (p < 0.0001) by the continent in which the MMSGT studies were performed. The continent with the highest reported frequency of PLGAs was Asia, with 3,702 of the 6,891 reported cases (53.7%), followed by America (24.2%) and Europe (19.1%), as shown in Table 4. The PLGA percentages also varied significantly by continent, with frequencies ranging from 3.9% in Asia to 20.0% in Oceania.

**Table 4:** The numbers and percentages of polymorphous low-grade adenocarcinomas in malignant minor salivary gland tumors by continent as described in the studies included in this review as described in the studies included in this review

**Continent**	**MMSGTs**		**PLGA**		**PLGA/MMSGTs**
	**n**	**%**	**n**	**%**	**%**
Africa	178	2.6%	14	3.2%	7.9%
America	1,668	24.2%	115	26.7%	6.9%
Asia	3,702	53.7%	144	33.4%	3.9%
Europe	1,313	19.1%	152	35.3%	11.6%
Oceania	30	0.4%	6	1.4%	20.0%
** Total**	**6,891**	**100.0**	**431**	**100.0**	**6.3**

MMSGTs: malignant minor salivary gland tumors; PLGA: polymorphous low-grade adenocarcinoma.

## Discussion

Analysis of the data from 57 epidemiological studies reflects a variety of methodologies, some examined all (major and minor) salivary gland tumors, while others examined only tumors of the minor glands but included benign and malignant tumors or even MMSGTs alone. This variability most likely reflects differences between the institutions from where most of the data were collected, such as hospitals and medical or dental schools. In other words, it does not reflect the real epidemiology of this tumor in these countries or continents, since they are a few isolated reports.

Nevertheless, it was possible to discern the PLGA percentages among the MMSGT cases, which was the aim of this study. We observed that PLGA rates have varied over the period studied and have most recently increased, most likely due to improved PLGA diagnostic accuracy. Over the last two study periods, the PLGA fraction has stabilized at a value that probably reflects a more accurate percentage of PLGAs among MMSGTs.

We also noted that the percentage varied by the continent where the studies were performed and by individual authors. Based on these results, we suggest that geographical differences alone cannot account for the varying incidence rates, such as occurs with Warthin tumor, which has a lower incidence in Africa, and with the lymphoepithelial carcinoma that has an evident predilection for Inuits (Eskimo), Chinese and Japanese [33,37,69,70]. Also based on these differences it is impossible to extracting other important data as the differences in ACC survival rates between Chinese and occidental data as recently demonstrated by Zhou et al. [71].

Despite our improved understanding of this entity over time, worldwide differences found amongst the studies indicate that diagnosing PLGA remains challenging, probably because histological and cytological criteria are not uniformly applied. Interestingly, this diagnosis does not appear in some of the series, which used the designation “adenocarcinoma” with no further definition, which raises the question of whether a tumor is actually an adenocarcinoma NOS, a PLGA or another entity.

Since the 1990s, many studies have attempted to develop a useful marker for PLGA or to differentiate it from other histologically similar tumors [72-75]. To date there has been no reliable molecular marker to distinguish PLGA from other MMSGTs [76]. The major research focus is currently on finding immunohistochemical differences between PLGA and adenoid cystic carcinoma (ACC), mainly in the cribriform histology, common to both tumors, which has been tirelessly attempted [77-87].

Controversy on this subject persists in the literature. Some authors believe that immunohistochemistry does not have any proven diagnostic value for identifying PLGA [6,78,88,89]. However, we do not share this opinion as we have successfully used immunohistochemistry in difficult cases or to confirm a histological diagnosis.

For diagnostic purposes, it is essential to characterize the morphology of the cell, the diversity of the histological tumor patterns and to recall that the PLGA cellular population exhibits a constant cytological appearance, despite a variety of growth patterns.

**Figure 1:**
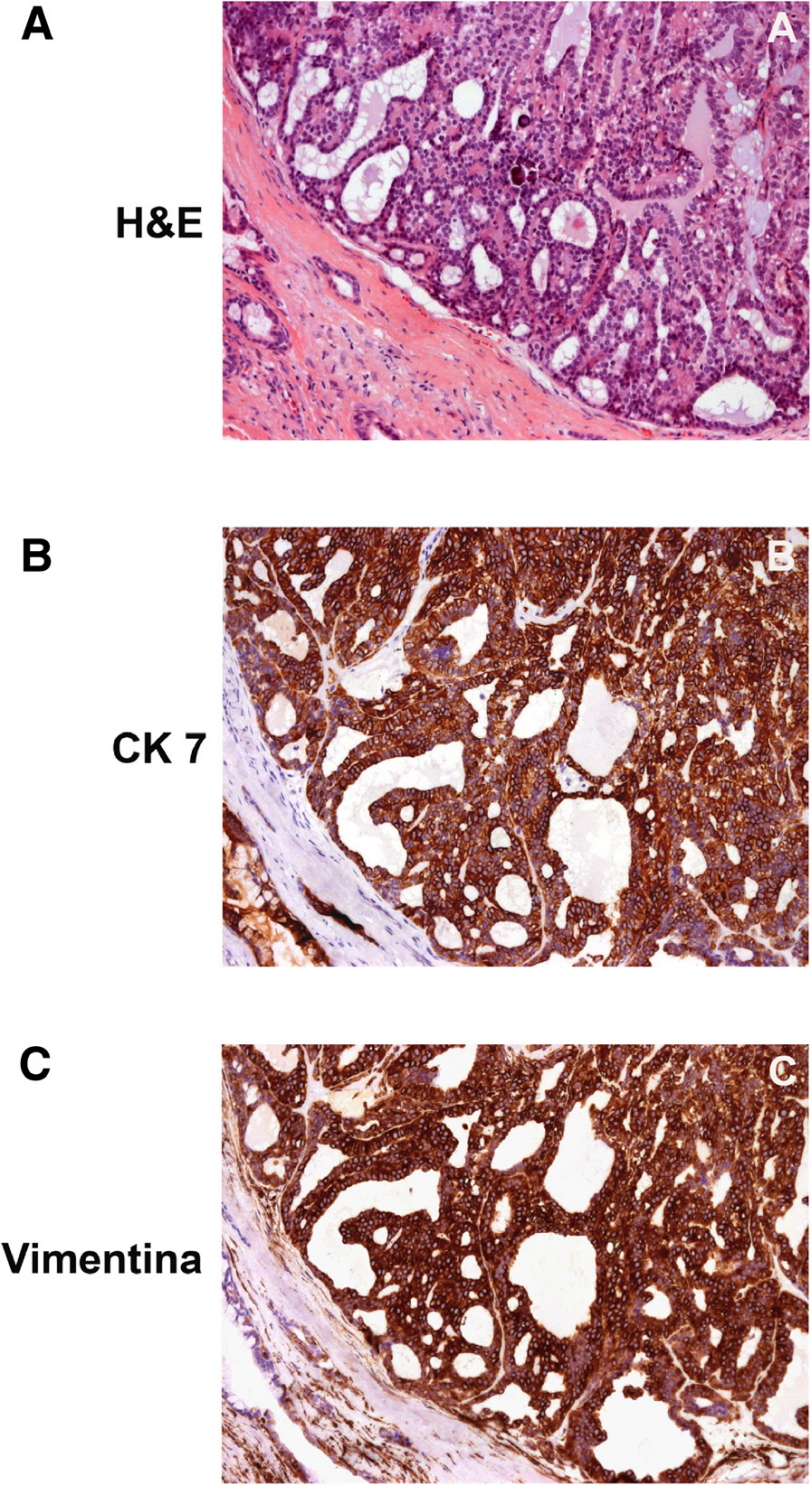
**The morphological and immunohistochemical aspects of PLGA.** 1**A**: H&E staining shows a tumor histology composed of uniform single-cell strands. Diffuse and strong CK7 (1**B**) and vimentin (1**C**) immunohistochemical positivity are shown.

In our experience it is important to note that tumor cytology and histology are usually sufficient for a final diagnosis. However, immunohistochemistry is valuable in unclear PLGA cases, however. Uniformly positive vimentin and CK 7 staining, except for the rare two-layer ducts, is sufficient for a final PLGA diagnosis (Figure 1). S100 is also positive in almost all of the cells, but this characteristic is only diagnostically supportive. When examining cytoskeleton filaments in salivary gland tumors, it is also important to observe which cells are positive for each protein, rather than simply indicating the percentage of tumors in a series that are positive for each marker. Using this information, the immunohistochemistry of the cytoskeleton filament contributes greatly to the diagnosis of salivary gland tumors, especially PLGAs.

## Competing interests

The authors declare that they have no competing interests.

## Authors’ contributions

VCA responsible for the conception and designed of research and wrote the most part of the manuscript. FPS and ABS responsible for collecting data. CT responsible for statistical analysis. NSA reviewed the manuscript. All authors read and approved the final manuscript.
